# Extracranial Internal Carotid Artery Tortuosity and Body Mass Index

**DOI:** 10.3389/fneur.2017.00508

**Published:** 2017-09-25

**Authors:** Hai-Feng Wang, Da-Ming Wang, Jun-Jie Wang, Li-Jun Wang, Jun Lu, Peng Qi, Shen Hu, Xi-Meng Yang, Kun-Peng Chen

**Affiliations:** ^1^Department of Neurosurgery, Beijing Hospital, National Center of Gerontology, Beijing, China; ^2^Graduate School of Peking Union Medical College, Beijing, China

**Keywords:** body mass index, obesity, blood flow, tortuosity, extracranial internal carotid artery

## Abstract

**Background:**

Extracranial internal carotid artery (eICA) tortuosity may trigger cerebral ischemia, and body mass index (BMI) is a measure of body mass based on height and weight. The main purpose of this study is to determine the influence of BMI on the tortuosity of eICA.

**Methods:**

A total of 926 carotid artery angiograms were performed in 513 patients, of which 116 cases and matched controls were selected. Arterial tortuosity was defined as simple tortuosity, kinking, or coiling. The severity of tortuosity was measured by tortuosity index, formula: [(actual length/straight-line length − 1) × 100].

**Results:**

BMIs were different between the two groups [tortuosity: 27.06 kg/m^2^ (SD 2.81 kg/m^2^) versus none: 23.3 kg/m^2^ (SD 2.78 kg/m^2^); *p* < 0.001]. BMI was independently and significantly associated with eICA tortuosity (odds ratio 1.59; 95% confidence interval, 1.35–1.86; *p* < 0.001). eICA tortuosity index is linearly associated with BMI (exponential coefficient β = 1.067, *p* < 0.001). The optimal predictive threshold of BMI for eICA tortuosity was 25.04 kg/m^2^. The physiological mechanism underlying the reasons why higher BMI has negative influence on extracranial carotid artery tortuosity may be an intra-abdominal hypertension caused by a much higher amount of body fat stored in visceral adipose tissue.

**Conclusion:**

Our result reveals a novel role for greater BMI on the presence of eICA tortuosity. For each increase in BMI of 1 kg/m^2^, there is a corresponding 1.59-fold increase in the risk of developing eICA tortuosity. The severity of eICA tortuosity increases linearly with increased BMI.

## Introduction

Tortuosity of the extracranial internal carotid artery (eICA) has been recorded since the nineteenth century (1). The incidence of eICA tortuosity ranges from 4 to 66%, while eICA tortuosity accounts for 4–16% symptomatic cerebrovascular insufficiency (2, 3). eICA tortuosity may lead to a reduction in artery pressure distal to the tortuosity, and the level of blood pressure drop is related to the severity of tortuosity (4). eICA tortuosity may also give rise to intermittent stenosis or occlusion by head rotation, trigger cerebral ischemia, and make intimal ulceration (5, 6). eICA tortuosity may increase the risk of carotid artery dissection by changing blood flow hemodynamics (7). A tortuous carotid artery is a source of danger in performing tonsillectomy, incising a peritonsillar abscess, or in the removal of adenoid neoplasms, because tortuous arteries may be incised by accident and leading to fatal hemorrhage (1, 8). Besides, eICA serves as the major gateway for endovascular interventions involving the intracranial vasculature such as treatments of aneurysms, stroke, vascular malformations, and tumors. Tortuosity of the eICA would hinder catheter-driven procedures.

The body mass is usually quantified by body mass index (BMI), defined as the body mass divided by the square of the body height, which categorizes a person as underweight, normal weight, overweight, or obese on its value. Obesity is a condition in which excess body fat has accumulated to the extent that it may have a negative effect on health (9). Fat stored within the abdominal cavity increases the abdominal static forces directly; the increased intra-abdominal pressure has many physiologic consequences, such as pushing diaphragm upward directly and raising mediastinum indirectly (10–12). Subsequently, the distance from aortic arch to skull base is shortened.

It has been demonstrated that the common carotid artery tortuosity is partially caused by elevation of the aortic arch. Since the origin is pushed cephalad, the carotid artery must buckle to accommodate the shortened distance between its proximal and distal ends (3). We hypothesize that shortened distance from aortic arch to skull base induces carotid artery to slide within the loose connective tissue of the carotid sheath against the skull base and results in eICA tortuosity. However, evidence of an association between BMI and eICA has not been reported yet.

The aim of this study was, therefore, to analyze the association between BMI and eICA tortuosity using data from a monocentric sample. We examined whether BMI was associated with eICA tortuosity and attempted to determine the optimal cutoff value of BMI for predicting tortuosity. We hypothesized that greater BMI would be associated with more severe eICA tortuosity.

## Materials and Methods

### Subjects

All procedures performed in studies involving human participants were in accordance with the ethical standards of the 1964 Helsinki Declaration and its later amendments or comparable ethical standards. This study was reviewed and approved by Beijing Hospital Ethics Committee. Due to the retrospective nature of this study, the requirement for informed consent from all subjects was waived.

This is an age- and sex-matched case–control study of patients undergoing diagnostic or therapeutic digital subtraction angiography (DSA) of carotid artery in our hospital. Between July 2014 and November 2015, data from all patients undergoing DSA of the carotid and vertebral arteries were prospectively recorded. All patients who underwent carotid angiogram, whatever the indication, were included to select cases and controls. Arterial tortuosity was defined as simple tortuosity, kinking, or coiling (3, 13). Patients with bilateral eICA arterial tortuosity were included as the cases. Patients had genetic connective tissue diseases were excluded. Based on these criteria, we included 116 consecutive eICA tortuosity patients. Age- and sex-matched controls were selected for each case and if there were multiple subjects with an identical age and sex for one tortuosity patient, the subject who underwent DSA on the closest date to the index patient was chosen as the control.

### Clinical Data

Baseline demographic and clinical information for all study subjects were prospectively collected, including age, sex, and vascular risk factors, such as hypertension (previous use of antihypertensive medication, systolic blood pressure > 140 mmHg or diastolic blood pressure > 90 mmHg on repeated measurement), diabetes mellitus (previous use of glucose-lowering medication or hemoglobin A1c ≥ 6.5%), dyslipidemia (previous use of lipid-lowering medication, low density lipoprotein cholesterol > 140 mg/dL, or total cholesterol > 220 mg/dL), habitual smoking (current or past regular smoking). Weight was measured by nurses to the nearest 0.1 kg using portable electronic scales, and height was measured to the nearest millimeter using a portable stadiometer. BMI was calculated as weight in kilograms divided by the square of height in meters.

### Angiographic Data

**Figure 1:**
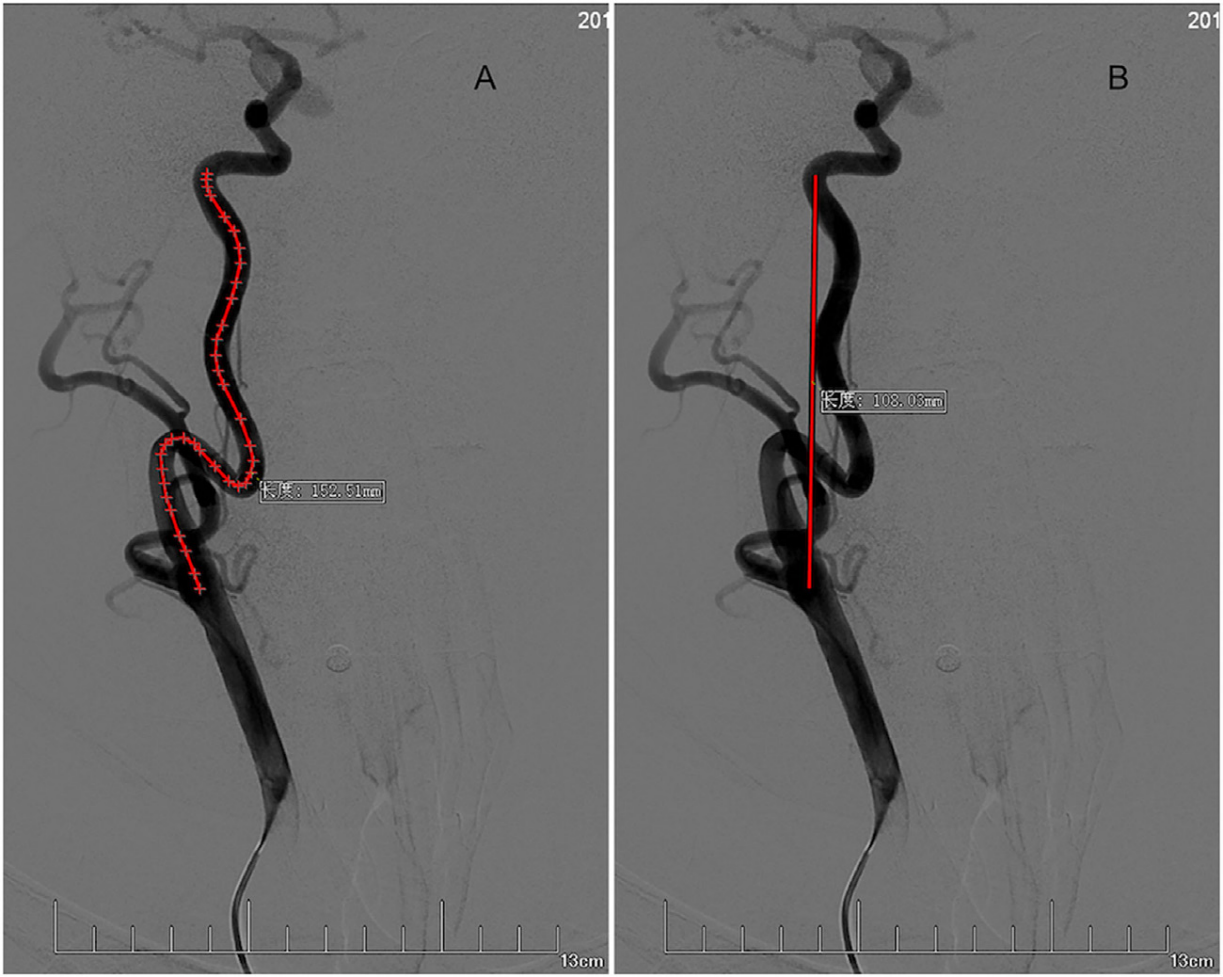
Measurement of tortuosity index. Arc length **(A)** and chord length **(B)** of right extracranial internal carotid artery (eICA) tortuosity is measured in the anteroposterior view of carotid artery angiography from the bifurcation point of the carotid artery to the distal end where ICA entered the carotid canal. For this right eICA, arc length = 152.51 mm, chord length = 108.03 mm. Tortuosity index = [(arc length/chord length − 1) × 100] = 41.

All DSA were performed with a biplane flat detector angiographies system (Allura Xper FD20, Philips, USA). Both eICAs in each patient were assessed by an investigator who blinded to the patient’s clinical information. For each subject, the anteroposterior views of both side carotid artery angiographies were used. The eICA was evaluated from its proximal end to distal end, the proximal end was the bifurcation point of the carotid artery, and the distal end was the point where ICA entered the carotid canal in the skull. The length between the two points including all the natural curvatures was used as the arc length, and the Euclidian distance between the two points was used as the chord length. All arc lengths and chord lengths were measured by PACS system (Neusoft system, Neusoft Corporation, Liaoning, China) (see Figure 1). The severity of tortuosity was measured by tortuosity index, which was calculated automatically as a ratio of the arc length of the blood vessel to the chord length between the two endpoints, formula: [(arc length/chord length − 1) × 100] (14).

### Interrater Reliability

To test the interrater reliability, the eICA tortuosity was assessed, measured and calculated by 2 independent investigators in 40 randomly selected study subjects (20 controls, 20 patients). Interrater reliability was calculated using Cohen kappa analysis and intraclass correlation coefficient.

### Statistical Analysis

All continuous variables with normal distribution were expressed as the mean and SD, if the distribution was skewed, the median and interquartile range (IQR) were used. Categorical variables were expressed as numbers and frequencies.

First, the demographic and clinical data were compared between cases and age- and sex-matched controls. The difference of normal distribution variables of cases and controls was tested by the paired Student’s *t*-test. We performed McNemar’s tests to determine whether there were differences in dichotomy variables (hypertension, diabetes mellitus, coronary artery disease, hyperlipidemia, and habitual smoker) between the two groups. Crude odds ratios (ORs) and 95% confidence interval (95% CI) were calculated using Miettinen and Nurminen method. We conducted conditional logistic regression to estimate the adjusted OR along with its 95% CI, vascular risk factors and the variables that reached a liberal statistical threshold of *p* < 0.2 were included in conditional logistic regression analysis.

Second, we performed receiver–operator characteristic curve analysis to determine the ability of BMI to predict tortuosity and to explore the optimal BMI threshold for eICA tortuosity.

Third, we conducted univariate analyses to look for associations between eICA tortuosity indexes and the outcomes of interest (BMI) or other covariates (hypertension, diabetes mellitus, coronary artery disease, hyperlipidemia, and habitual smoking). Histogram and Shapiro–Wilk test were used to determine whether the average, right eICA tortuosity index (RICATI) and left eICA tortuosity index (LICATI) were well-modeled normal distribution or skewed distribution. The correlations between eICA tortuosity index and continuous variables or dichotomy variables were calculated by Spearman’s correlation coefficient. Any test results reaching a liberal statistical threshold of *p* < 0.2 for each comparison were then entered multivariable linear regression model to identify independently predictive factors for tortuosity. Vascular risk factors were forced to enter into the multivariate linear regression model to identify independently predictive factors for tortuosity. Then, eICA tortuosity index was converted into natural logarithmic equivalent value for statistical analysis. Variance inflation factor were calculated to quantify the severity of multicollinearity in the multivariate linear regression model. We conducted histogram and Shapiro–Wilk test to determine the residual distribution, and we found the residuals were well-modeled normal distribution. Wilcoxon signed-rank test was performed to compare the eICA tortuosity indexes of the two sides.

Data analysis was performed using the statistical package for social sciences version 24.0 (SPSS, Inc., Chicago, IL, USA) and R Statistical Software (Version 3.4.0, 2017, Vienna, Austria). For all final models, statistical significance was set at two-tailed *p* < 0.05.

## Results

### Subjects

A total of 926 carotid artery angiograms were performed in 513 patients. Among those patients, 116 (22.6%) bilateral eICA tortuosity cases fulfilled inclusion criteria, and 116 age- and sex-matched controls were selected for each case. None of the patients were diagnosed with an overt connective tissue disorder. The mean age of cases or controls was 62.41 ± 0.81 years, and 62 cases or controls (53.4%) were men (see Table 1).

**Table 1:** Baseline characteristics of the subjects.

Variable	Cases (*n* = 116)	Controls (*n* = 116)	*p*-Value
Age, mean ± SD, years	62.41 ± 0.81	62.41 ± 0.81	
Male sex, *n* (%)	62 (53.4%)	62 (53.4%)	
BMI, mean ± SD	27.06 ± 2.81	23.3 ± 2.78	<0.001^†^
Hypertension, *n* (%)	87 (75%)	69 (59.5%)	0.018^†^
DM, *n* (%)	33 (28.4%)	28 (24.1%)	0.533
Dyslipidemia, *n* (%)	80 (69%)	77 (66.4%)	0.755
CAD, *n* (%)	28 (24.1%)	31 (26.7%)	0.749
Smoking, *n* (%)	48 (41.4%)	42 (36.2%)	0.511

*Results are expressed as a number (%) or mean ± SD, comparison between BMI using paired *t*-tests; McNemar’s tests used in dichotomy characters*.

*^†^Significant variables*.

*DM, diabetes mellitus; CAD, coronary artery disease; BMI, body mass index*.

### Clinical Factors Associated With eICA Arterial Tortuosity

Patients with bilateral eICA tortuosities were compared with the matched controls who did not have any eICA tortuosity (see Table 1). During univariate analysis, eICA tortuosity patients were more likely to present with a higher BMI, as compared with controls (tortuosity: 27.06 ± 2.81 kg/m^2^ versus none: 23.3 ± 2.78 kg/m^2^; *p* < 0.001). Patients with eICA tortuosities were more likely to have hypertension (tortuosity: 75% versus none: 59.5%; *p* = 0.018). There were no significant differences in other factors (diabetes mellitus, dyslipidemia, coronary disease, and habitual smoking). By conditional logistic regression, BMI (OR 1.59; 95% CI, 1.35–1.86; *p* < 0.001) was independently and significantly associated with the presence of eICA tortuosity (see Table 2).

**Table 2:** Characters’ ORs for extracranial internal carotid artery tortuosity.

Characters	Crude OR (95%)	Unadjusted *p*-value	Adjusted OR (95%)	Adjusted *p*-value
BMI	1.576 (1.346–1.845)	<0.001	1.585^a^ (1.349–1.863)	<0.001^†^
Hypertension	2.059 (1.168–3.63)	0.018	1.092 (0.495–2.410)	0.827
DM	1.278 (0.691–2.364)	0.533	0.757 (0.291–1.971)	0.569
Dyslipidemia	1.158 (0.652–2.057)	0.755	0.718 (0.301–1.714)	0.455
CAD	0.857 (0.457–1.608)	0.749	0.931 (0.366–2.366)	0.881
Smoking	1.313 (0.508–3.385)	0.511	0.983 (0.412–2.346)	0.969

*Data are presented as OR (95% confidence interval). Pearson correlation coefficient for normal distribution variables or McNemar’s tests for dichotomy variables were used to calculate crude OR. Conditional logistic regression was used to calculate adjusted OR*.

*^a^Data are presented as OR per unit increase in BMI (kg/m^2^)*.

*^†^Significant variables*.

*DM, diabetes mellitus; CAD, coronary artery disease; OR, odds ratio; BMI, body mass index*.

**Figure 2:**
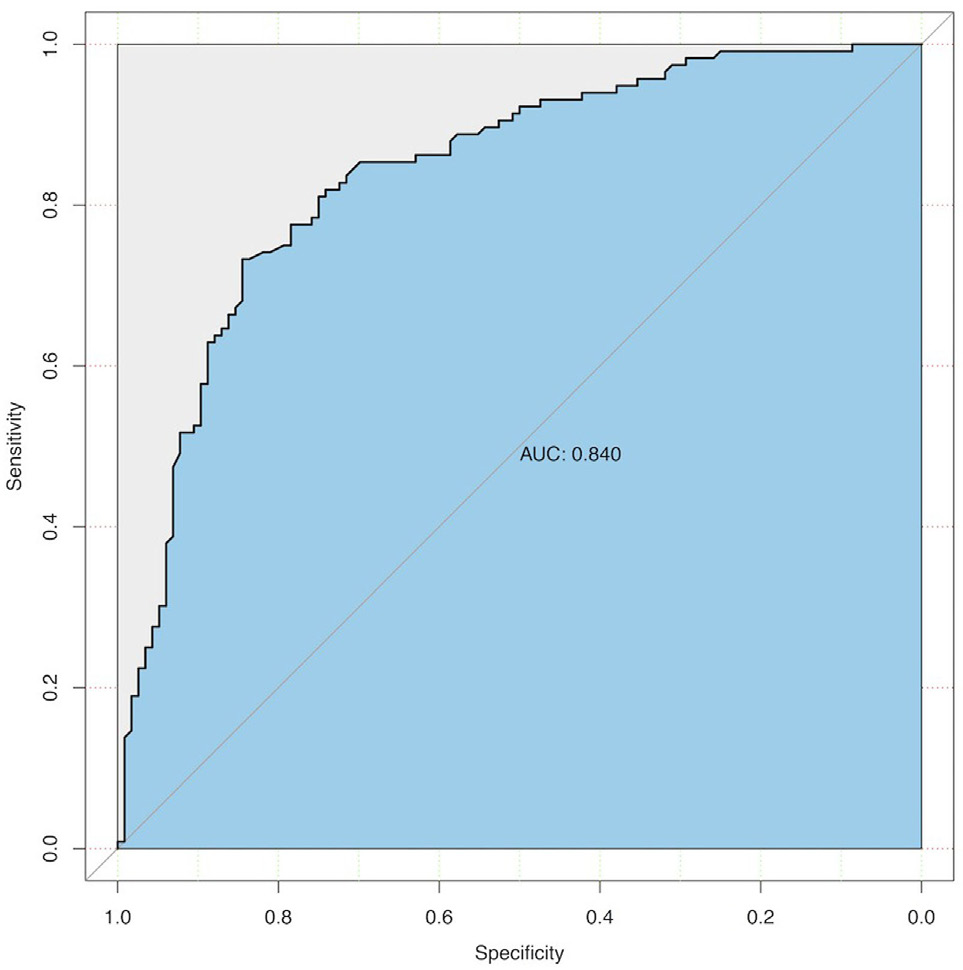
ROC curve of the predictive value of BMI for eICA tortuosity. Increasing BMI was predictive of eICA tortuosity, area under the curve = 0.84; 95% confidence interval, 0.79–0.89; *p* < 0.001. The optimal predictive threshold of BMI for eICA tortuosity was 25.04 kg/m^2^. eICA, extracranial internal carotid artery; BMI, body mass index.

To identify accurate threshold for BMI predicting eICA tortuosity, we constructed receiver–operator characteristic curves. Increased BMI was associated with higher risk of eICA tortuosity (area under the curve, 0.84; 95% CI, 0.79–0.89; *p* < 0.001). The optima predictive threshold of BMI for eICA tortuosity was 25.04 kg/m^2^ (sensitivity: 78%; specificity: 78%; see Figure 2).

### Tortuosity Index and Associated Clinical Factors

**Figure 3:**
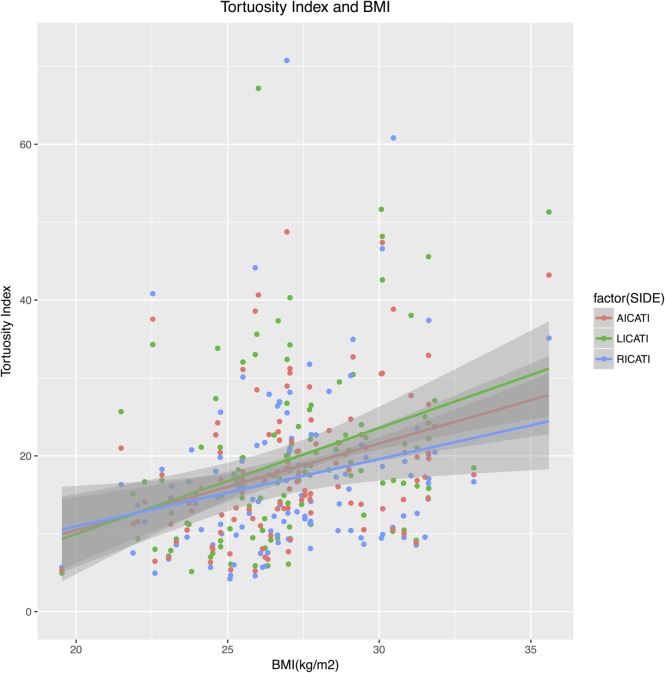
The severity of eICA tortuosity increased with increasing BMI. Correlation between AICATI and BMI, *n* = 116, ρ = 0.386, *p* < 0.001. Correlation between LICATI and BMI, *n* = 116, ρ = 0.394, *p* < 0.001. Correlation between RICATI and BMI, *n* = 116, ρ = 0.270, *p* = 0.003. Gray area around the regression line represents the 95% confidence interval. eICA, extracranial internal carotid artery; AICATI, average eICA tortuosity index; LICATI, left eICA tortuosity index; RICATI, right eICA tortuosity index; BMI, body mass index.

The median average eICA tortuosity index (AICATI) of the bilateral eICA was 16.82 (IQR, 12.48–22.52). Histogram and Shapiro–Wilk test demonstrated AICATI was right-skewed distribution. In correlation analysis of average bilateral eICA tortuosity index with clinical factors, spearman’s correlation coefficient was used, male sex (ρ = −0.27, *p* = 0.004) and BMI (ρ = 0.39, *p* < 0.001) were significantly correlated with tortuosity index (see Table 3). The variables that reached the statistical threshold of *p* < 0.2 were put into the multivariate linear regression model. However, all the rest variables were vascular risk factors; they were forced to enter into the multivariate linear regression model. After adjusting for age, gender, hypertension, diabetes mellitus, dyslipidemia, coronary disease, and habitual smoking, natural logarithmic AICATI remained associated with BMI (β = 0.07, *p* < 0.001, incremental *R*^2^ = 0.15); and the exponentiated coefficient exp(β) for BMI was 1.07. Adjusting for all other covariates, male sex (β = −0.22, *p* = 0.008, incremental *R*^2^ = 0.05) was significantly associated with natural logarithmic average tortuosity index, and exp(β) for male was 0.80. Variance inflation factor of both gender and BMI was 1.004, multicollinearity could be safely ignored. The association between BMI and average bilateral eICA tortuosity index was presented graphically in figure (see Figure 3).

**Table 3:** Associations between average eICA tortuosity index (AICATI) and subjects’ characteristics.

Characteristics	Spearman’s rank correlation coefficient		Multiple linear regression		

	ρ^a^	*p*-Value	β^b^	*p*-Value	VIF
Age (years)	0.105	0.262	0.052	0.559	1.092
Male sex	−0.267	0.004	−0.222	0.008	1.004
BMI (kg/m^2^)	0.386	<0.001	0.065^c^	<0.001	1.004
Hypertension	0.151	0.106	0.036	0.681	1.095
DM	0.076	0.416	0.046	0.591	1.051
Dyslipidemia	0.115	0.218	0.046	0.594	1.029
CAD	−0.109	0.245	−0.041	0.634	1.016
Smoking	0.033	0.723	0.186	0.059	1.379

*^a^Spearman’s rank correlation coefficient between AICATI and each variable; ρ: Spearman’s correlation coefficient*.

*^b^Multiple linear regression analysis, *β*: parameter estimate; eICA tortuosity index was converted into natural logarithmic equivalent value for statistical analysis; exponentiated coefficient exp(*β*) for BMI was 1.067, exp(*β*) for male was 0.801*.

*^c^Data are presented as natural logarithmic eICA tortuosity index per unit increase in BMI (kg/m^2^)*.

*DM, diabetes mellitus; CAD, coronary artery disease; eICA, extracranial internal carotid artery; VIF, variance inflation factor; BMI, body mass index*.

The median eICA tortuosity indexes of the left and right eICA were 17.59 (IQR 11.25–22.68) and 14.52 (IQR 9.85–20.58), respectively. In Wilcoxon’s signed-rank test, the difference between LICATI and RICATI was statistically significant (*p* < 0.001). To ensure that BMI had an impact on both side eICA tortuosities, we performed a further analysis of eICA tortuosity indexes of each side and associated clinical factors. Spearman’s correlation coefficient was used in the correlation analysis, male sex (ρ = −0.22, *p* = 0.02) and BMI (ρ = 0.39, *p* < 0.001) were significantly correlated with LICATI (see Table 4). In multivariate linear regression model, holding all other variables at any fixed value, natural logarithmic LICATI remained associated with BMI (β = 0.08, *p* < 0.001, incremental *R*^2^ = 0.16), exp(β) for BMI was 1.08. Holding other variables, male sex (β = −0.20, *p* = 0.03, incremental *R*^2^ = 0.03) was significantly associated with natural logarithmic left tortuosity index, exp(β) for male was 0.82 (see Table 4). In the correlation analysis, male sex (ρ = −0.21, *p* = 0.023) and BMI (ρ = 0.27, *p* = 0.003) were significantly correlated with RICATI. In multivariate linear regression model, BMI [β = 0.05, *p* = 0.003, incremental *R*^2^ = 0.08; exp(β) = 1.05] and male sex [β = −0.23, *p* = 0.02, incremental *R*^2^ = 0.04; exp(β) = 0.80] were significantly and independently associated with right tortuosity index (see Table 4). Variance inflation factor of both gender and BMI was also 1.004, multicollinearity could be safely ignored. The association between BMI and each side eICA tortuosity index was presented graphically in figure (see Figure 3).

**Table 4:** Associations between eICA tortuosity index and subjects’ characteristics.

Characteristics	Right eICA					Left eICA				
	ρ^a^	*p*-Value	β^b^	*p*-Value	VIF	ρ^a^	*p*-Value	β^b^	*p*-Value	VIF
Age (years)	0.138	0.141	0.108	0.241	1.092	0.068	0.467	−0.015	0.868	1.092
Male sex	−0.211	0.023	−0.227	0.021^†^	1.004	−0.215	0.021	−0.197	0.032^†^	1.004
BMI (kg/m^2^)	0.270	0.003	0.053^c^	0.003^†^	1.004	0.394	0.000	0.075^c^	<0.001^†^	1.004
Hypertension	0.113	0.227	0.003	0.976	1.095	0.161	0.084	0.076	0.394	1.095
DM	0.012	0.901	0.007	0.937	1.051	0.162	0.082	0.094	0.277	1.051
Dyslipidemia	0.097	0.301	0.033	0.712	1.029	0.165	0.076	0.071	0.407	1.029
CAD	−0.092	0.326	−0.060	0.501	1.016	−0.043	0.649	0.011	0.902	1.016
Smoking	−0.069	0.460	0.200	0.053	1.379	−0.026	0.780	0.113	0.256	1.379

*^a^Spearman’s rank correlation coefficient between eICA tortuosity index and each variable; ρ: Spearman’s correlation coefficient*.

*^b^Multiple linear regression analysis, *β*: parameter estimate; eICA tortuosity index was converted into natural logarithmic equivalent value for statistical analysis. Right eICA tortuosity: exponentiated coefficient exp(*β*) for BMI was 1.054, exp(*β*) for male was 0.797. Left eICA tortuosity: exponentiated coefficient exp(*β*) for BMI was 1.078, exp(*β*) for male was 0.821*.

*^c^Data are presented as natural logarithmic tortuosity index per unit increase in BMI (kg/m^2^)*.

*^†^Significant variables*.

*DM, diabetes mellitus; CAD, coronary artery disease; eICA, extracranial internal carotid artery; VIF, variance inflation factor; BMI, body mass index*.

### Interrater Reliability

We conducted Cohen kappa analysis in 40 randomly selected study subjects (20 controls, 20 cases) to test interrater reliability. Result from the Cohen kappa analysis was excellent (κ = 0.93, *p* < 0.001). eICA tortuosity index of the 20 cases who were randomly chosen for interrater reliability analysis, 40 extracranial internal carotid arteries, ranged from 6 to 68. Interclass correlation coefficient for eICA tortuosity index comparison between the two observers was 0.98 (*p* < 0.001).

## Discussion

We conducted an age- and sex-matched case–control study to determine whether greater BMI was a risk factor for eICA tortuosity. The results of this study demonstrate that increased BMI correlates with higher risk of eICA tortuosity, and tortuosity index elevates linearly with BMI. For each increase in BMI of 1 kg/m^2^, there is a corresponding 1.59-fold increase (OR: 1.59, 95% CI: 1.35–1.86, *p* < 0.001) in the risk of developing eICA tortuosity. For each increase in BMI of 1 kg/m^2^, there is a corresponding 0.067 increase in AICATI. If a patients’ BMI increases from 20 to 30 kg/m^2^, AICATI will increase 0.67; it means that the actual length of eICA is 0.67-fold longer than straight length from the bifurcation point of the carotid artery to the point where eICA entered the carotid canal in the skull. Because eICA tortuosity may lead to a reduction in artery pressure and the level of blood pressure drop is related to the severity of tortuosity (4). It means obesity itself may cause insufficient blood flow to the brain. The optimal cutoff value of BMI for eICA tortuosity is 25.04 kg/m^2^, being approximate to the threshold value for overweight. These findings suggest that overweight or obesity may be a risk factor for eICA tortuosity.

The prevalence of eICA tortuosity in our study is consistent with those of previous reports; the incidence of tortuosity has been calculated at 4–66% in adult (3, 15–19). The variability of incidence may be caused by difference diagnostic methodology and patients’ inclusion criteria, but in aggregate they concur to indicate that this is a rather frequent finding.

It is still controversial whether eICA tortuosity is related to gender. Our results show, holding all other variable at any fixed value in multivariate analysis, female is significantly and independently correlated with tortuosities index. Most of the previous studies showed a more frequent of eICA tortuosity in female sex (18, 20, 21). But there were no studies to test the correlation between tortuosity index and gender. The results of our study demonstrate the severity of tortuosity is associated with gender. Because the controls were sex-matched, we could not compare the prevalence rate of eICA tortuosity between sexes. Our results do not consist with studies that demonstrate eICA tortuosity is correlated with vascular risk factors, such as hypertension, hyperlipidemia, diabetes, and atherosclerotic diseases (17, 20), but accord with studies that show there is no correlation between eICA tortuosity and vascular risk factors (2, 16, 18).

Our results herein also show that eICA tortuosity index is right-skewed distribution; it means that most of the eICA tortuosity patients have minor eICA tortuosity. Although there are no previous reports, this can be easily understood. The threshold for eICA tortuosity is 25.04 kg/m^2^, the value is appropriated with the threshold of overweight 25 kg/m^2^. Thus, the distribution of tortuosity should like the section of BMI distribution from overweight to obesity; it is a skewed distribution because BMI is normal distribution. This finding confirms the linear correlation between BMI and eICA tortuosity.

The physiological mechanism underlying the reasons why BMI has influence on eICA tortuosity may be an intra-abdominal hypertension. Obesity generally means a much higher amount of body fat stored in body, and fat located in visceral adipose tissue increase intra-abdominal static pressure. Increased intra-abdominal static pressure has many physiologic consequences: increases in central venous pressure, pulmonary capillary wedge pressure, systemic vascular resistance, peak airway pressure, intrapleural pressure, renal vein pressure and decreases in venous return, cardiac output, visceral blood flow, renal blood flow, glomerular filtration rate, and abdominal wall compliance. Increased intra-abdominal pressure can also push diaphragm upward directly (10, 11). Since the diaphragm moves cephalad, corresponding to a raised mediastinum that include heart, aortic arc, brachiocephalic artery, and left common carotid artery. Carotid arteries are surrounded by loose connective tissue within carotid sheath, in which artery can slide. Elevated mediastinum gives rise to a force of elevation acting upon and moving arteries cranially. As the force of elevation transmitting upward, brachiocephalic artery and common carotid artery raise, the distance from eICA origin to the end is shortened. The straight-line distance is shortened, but the actual length of artery remains the same, so the tortuosity happens.

The left eICA tortuosity is more severe than right, the difference is probably owing to eICA proximal arteries. Brachiocephalic artery is the first and largest branch of the aortic arch; the right common carotid artery arising from the brachiocephalic artery passes directly upward in the neck and divides to form the external and internal carotid arteries. Because of the angle between brachiocephalic artery and right common carotid artery, force of elevation transmitting through brachiocephalic artery lose more force near the bifurcation of right common carotid artery and subclavian artery. The lost force acts upon proximal segment of common carotid artery and causes a relatively severe tortuosity at the site of the origin of the right common carotid artery (22–24). Therefore, less force of elevation can eventually transmit to eICA, which results in right eICA tortuosity less severe than the left one. On the left side, the common carotid artery arises from aortic arch and has an approximate straight course between its origin and the skull. Force of elevation can transmit upward directly and lose little, eventually more force transmit to eICA and bring about more severe tortuosity.

The eICA on each side has a fairly similar course. From its origin at the bifurcation of the common carotid artery, it first passes laterally and then posteromedially to the external carotid artery before continuing almost perpendicularly upward to the carotid canal (3). Because bilateral eICA arteries have the similar course, blood flow volume, diameter, structure of vessel wall, surrounding tissues, and supplies blood to equivalent area of the brain. The difference of tortuosity indexes between two sides should attribute to its proximal artery, and this confirms our hypothesis that the eICA tortuosity was caused by elevated mediastinum and force of elevation transmitting through proximal artery to eICA.

Up to date, the natural history of eICA tortuosity is not well known, although we hypothesize high BMI as one possible reason of eICA tortuosity, eICA tortuosity remains a complex poorly understood phenomenon. In our multivariate linear regression model of tortuosity index and associated clinical factors, the incremental *R*^2^ of BMI is equal to 0.15; it can be interpreted as follows: 15% of the eICA tortuosity index can be explained by BMI, the remaining 85% can not be attributed to BMI. There are multiple factors involved in the etiology of eICA tortuosity; mechanical factors alone cannot explain all causes why eICA tortuosity happens. There have been several hypotheses about why arterial tortuosity happens. Higher BMI and the related components of the metabolic syndrome could have many physiological and ultrastructural alterations at the tissue level especially in the blood vessels. Repeated and prolonged exposure to periods of high flow and low wall shear stress affects arterial remodeling along the length of an artery and cause tortuosity (25). Cervical artery tortuosity is associated with intracranial aneurysm and cervical artery dissection, vertebral artery tortuosity is associated with genetic connective tissue disorders, these support the concept that tortuosity may reflect a diffuse arteriopathy weakening the arterial wall, ultimately leading to increased vascular tortuosity (7, 14, 26). The histological examination of eICA tortuosity specimens showed a reduction of elastic fibers and muscular cells with a compensative increase of connective fibers, which support the hypothesis that eICA tortuosity is metaplastic transformation (2). Prevalence of carotid artery tortuosity showed no increase with age, which imply eICA tortuosity is embryological origin (16), but the result may also be interpreted as the diagnostic method of color Doppler ultrasonography used in this study was not sensitive enough to find the difference between normal and minor tortuosity artery, as most of eICA tortuosities were minor, so false negative result occurs.

This study needs to be considered alongside limitations that may have confounded the results. First, we evaluate the severity of tortuosity on the anteroposterior views of both side carotid artery angiographies; however, eICA tortuosity may occur in either the coronal or the sagittal plane due to the three-dimensional nature of vascular tortuosity (27). Because the tortuosity in sagittal plane cannot be detected on the anteroposterior views of carotid artery angiographies, the real correlation of eICA tortuosity and BMI may be stronger than what we discover. Second, our study had a retrospective design with inherent limitation leading to potential ascertainment bias, no matter the case–control study was well matched, there might be selection bias. Third, we hypothesize that higher BMI causes eICA tortuosity by intra-abdominal hypertension, elevated diaphragm and mediastinum, but we do not direct proofs. We demonstrate obesity is related to eICA tortuosity, but direct proofs are warranted to validate our finding, such as animal study or direct evidence from imaging. Although now widely used as a measure of obesity, the numerator in the calculation of BMI, body weight, does not distinguish between fat mass and lean tissue mass. A more valid and precise measure of the distribution of body fat, such as measurements obtained with modern investigative methods, such as CT, MRI, or dual-energy X-ray absorptiometry, would be preferred. Although BMI is not an ideal index of obesity, it is used in this study, as in many other studies, because it is a practicable index for large-scale studies. The correlation between obesity and eICA tortuosity may be underestimated by use of BMI as measurement.

## Conclusion

Our result reveals a novel role for greater BMI on the presence of eICA tortuosity. For each increase in BMI of 1 kg/m^2^, there is a corresponding 1.59-fold increase in the risk of developing eICA tortuosity. To the best of our knowledge, this is also the first study that demonstrates the severity of eICA tortuosity responses linearly to BMI, and eICA tortuosity index is right-skewed distribution. We also found left eICA tortuosity is more severe than the right one. These three findings confirm the association between eICA tortuosity and BMI and support our hypothesis that higher BMI causes eICA tortuosity by intra-abdominal hypertension, elevated diaphragm, and mediastinum. Further studies are merited to validate our finding, such as animal study or direct evidence from imaging.

## Ethics Statement

This study was carried out in accordance with the recommendations of “Beijing Hospital Ethics Committee.” Due to the retrospective nature of this study, the requirement for informed consent from all subjects was waived.

## Author Contributions

H-FW drafted the manuscript and performed data collection and data analysis. D-MW participated in the design of this study and helped to check the manuscript. J-JW performed data collection. L-JW, JL, PQ, SH, X-MY, and K-PC performed the operations in this study.

## Conflict of Interest Statement

The authors declare that the research was conducted in the absence of any commercial or financial relationships that could be construed as a potential conflict of interest.
